# Discrimination Between Human Leukocyte Antigen Class I-Bound and Co-Purified HIV-Derived Peptides in Immunopeptidomics Workflows

**DOI:** 10.3389/fimmu.2018.00912

**Published:** 2018-04-27

**Authors:** Thomas Partridge, Annalisa Nicastri, Anna E. Kliszczak, Louis-Marie Yindom, Benedikt M. Kessler, Nicola Ternette, Persephone Borrow

**Affiliations:** ^1^Nuffield Department of Medicine, University of Oxford, Oxford, United Kingdom; ^2^Nuffield Department of Medicine, Target Discovery Institute, University of Oxford, Oxford, United Kingdom; ^3^The Jenner Institute, Target Discovery Institute Mass Spectrometry Laboratory, University of Oxford, Oxford, United Kingdom

**Keywords:** HIV, major histocompatibility complex, human leukocyte antigen, epitope, antigen presentation, immunopeptidomics, mass spectrometry

## Abstract

Elucidation of novel peptides presented by human leukocyte antigen (HLA) class I alleles by immunopeptidomics constitutes a powerful approach that can inform the rational design of CD8^+^ T cell inducing vaccines to control infection with pathogens such as human immunodeficiency virus type 1 (HIV-1) or to combat tumors. Recent advances in the sensitivity of liquid chromatography tandem mass spectrometry instrumentation have facilitated the discovery of thousands of natural HLA-restricted peptides in a single measurement. However, the extent of contamination of class I-bound peptides identified using HLA immunoprecipitation (IP)-based immunopeptidomics approaches with peptides from other sources has not previously been evaluated in depth. Here, we investigated the specificity of the IP-based immunopeptidomics methodology using HLA class I- or II-deficient cell lines and membrane protein-specific antibody IPs. We demonstrate that the 721.221 B lymphoblastoid cell line, widely regarded to be HLA class Ia-deficient, actually expresses and presents peptides on HLA-C*01:02. Using this cell line and the C8166 (HLA class I- and II-expressing) cell line, we show that some HLA class II-bound peptides were co-purified non-specifically during HLA class I and membrane protein IPs. Furthermore, IPs of “irrelevant” membrane proteins from HIV-1-infected HLA class I- and/or II-expressing cells revealed that unusually long HIV-1-derived peptides previously reported by us and other immunopeptidomics studies as potentially novel CD8^+^ T cell epitopes were non-specifically co-isolated, and so constitute a source of contamination in HLA class I IPs. For example, a 16-mer (FLGKIWPSYKGRPGNF), which was detected in all samples studied represents the full p1 segment of the abundant intracellular or virion-associated proteolytically-processed HIV-1 Gag protein. This result is of importance, as these long co-purified HIV-1 Gag peptides may not elicit CD8^+^ T cell responses when incorporated into candidate vaccines. These results have wider implications for HLA epitope discovery from abundant or membrane-associated antigens by immunopeptidomics in the context of infectious diseases, cancer, and autoimmunity.

## Introduction

CD8^+^ T cells are able to recognize pathogen-infected cells by engaging human leukocyte antigen (HLA) class I molecules in complex with short (typically 8–11 amino acids) peptides on the cell surface through rearranged T cell receptors. The diverse repertoire of thousands of individual peptides presented on HLA class I is known as the “immunopeptidome.” Following recognition of cognate peptide (epitope)–HLA class I complexes, CD8^+^ T cells can then induce apoptotic or lytic death of the target cell by exocytosing granules containing granzyme and perforin and/or ligating death receptors, as well as being triggered to release cytokines such as TNF-α and IFN-γ.

A body of evidence suggests that CD8^+^ T cells are important in controlling the replication of viruses such as human immunodeficiency virus type 1 (HIV-1). First, the expansion of CD8^+^ T cells in acute HIV-1 infection (AHI) is temporally associated with control of viremia (1, 2); furthermore, CD8^+^ T cells rapidly drive selection for escape mutations in HIV-1 during AHI (3–5). Elite controllers, who maintain low viral loads in the absence of antiretroviral therapy, maintain polyfunctional CD8^+^ T cell responses to HIV-1 epitopes throughout infection (6). In addition, certain HLA class I alleles are linked to fast or slow disease progression (7–10). Finally, when CD8^+^ T cells are depleted in the macaque simian immunodeficiency virus (SIV) challenge model, control of viremia during acute infection is hindered (11). Notably, immunization of rhesus macaques with a live attenuated rhesus cytomegalovirus vector encoding SIV antigens induced strong effector CD8^+^ T cell responses against novel SIV epitopes and remarkably, 50% of immunized animals were able to clear SIV infection (12, 13). Therefore, definition of the immunopeptidome presented by infected cells for recognition by HIV-1-specific CD8^+^ T cells holds great importance for the design of CD8^+^ T cell-inducing vaccines against HIV-1.

Traditionally, human CD8^+^ T cell epitopes have been defined by synthesis of overlapping peptide pools followed by screening to identify peptides that are recognized by patient peripheral blood mononuclear cells (PBMC) *ex vivo* (14). However, this method does not reveal peptides against which T cell responses were not elicited in the donors screened, and epitope responses may be missed or overestimated as a result of the artificial peptide stimulation. To overcome this problem, prediction algorithms have been developed to identify class I-binding peptides (15); however, their accuracy can be poor for less well-characterized HLA alleles. In recent years, advances in the sensitivity of state-of-the-art liquid chromatography tandem mass spectrometry (LC-MS/MS) instrumentation have revealed thousands of naturally presented HLA-restricted peptides from complex immunopeptidomes in a single measurement (16). Typically, HLA class I complexes are isolated from the cells or tissue of interest by immunoprecipitation (IP), dissociated at low pH then peptides are purified for sequencing by LC-MS/MS. Alternatively, peptides bound to HLA class I are isolated directly from the cell surface by mild acid elution. These MS-based immunopeptidomics methodologies have shown great utility for epitope discovery in the context of infectious diseases (17, 18), cancer neoantigens (19–22), HLA-associated drug sensitivities (23), and targets of autoreactive T cells (24).

Recent immunopeptidomic studies have investigated the repertoire of HIV-1 peptides presented by CD4^+^ cell lines or primary cells infected *in vitro* with HIV-1 (25–27). These studies were successful in identifying multiple previously unknown HIV-1-derived epitopes of potential utility for vaccine design. Furthermore, these studies yielded an unexpected abundance of nested sets of peptides extended at the N- or C-termini, as well as unusually long peptide species predominantly derived from HIV-1 Gag p15. Intriguingly, some of these extended peptides were identified in all three studies published to date, despite differences in the HLA types of cells and methodologies used. Although some of these long HIV-1 peptides were recognized by T cells from some HIV-infected donors in IFNγ ELISPOT assays, no conclusive evidence that these are optimal HLA class I-restricted peptides has been shown. Furthermore, the measured binding affinity of many of these long peptides to HLA class I was found to be very low *in vitro* (26). Unusually long (>13 amino acids) and low affinity peptides binding promiscuously across diverse donor HLA class I types would be unprecedented.

The HLA IP procedure is thought to be highly specific, despite a substantial loss of HLA class I complexes at this step (28). However, the extent of contamination of class I-bound peptides identified using HLA IP-based immunopeptidomics workflows with peptides from other sources has not been formally evaluated. Here, the specificity of the IP-based immunopeptidomics methodology for identifying self/HIV-1-derived HLA class I-restricted peptides was examined through the use of antibodies directed against membrane proteins and HLA class I/II negative cell lines. We hypothesized that the HLA class I IP procedure results in low-level co-isolation of non-specific peptides, which may be erroneously assigned as HLA class I-restricted. We show that (1) HLA class II-derived peptides co-isolate non-specifically in HLA class I IPs; (2) extended peptides derived from the abundant HIV-1 Gag protein co-purify non-specifically in HLA class I IPs; and (3) the B lymphoblastoid cell line 721.221 widely believed to be deficient in classical HLA class I alleles actually expresses and presents peptides on HLA-C*01:02.

## Materials and Methods

### Cell Culture

C8166 cells were obtained from the National Institute for Biological Standards and Control (NIBSC) Centre for AIDS Reagents (CFAR). The HLA class I-deficient 721.221 cell line expressing CD4 (CD4.221) was a kind gift from Professor Masafumi Takaguchi (Kumamoto University, Japan). T2 cells [hemizygous for chromosome 6, transporter associated with antigen processing (TAP)-deficient, HLA class II-deficient] were obtained from the American Type Culture Collection. C8166, CD4.221, and T2 cells were routinely cultured in RPMI 1640 medium containing 10% fetal bovine serum (FBS), 100 μg/ml penicillin, 100 IU/ml streptomycin, and 10 mM HEPES (R10) at 37°C with 5% CO_2_. Hybridomas used for antibody production were kindly provided by Alastair Waugh at the Human Immunology Unit of the Weatherall Institute for Molecular Medicine (WIMM). W6/32 hybridoma cells were cultured in a CELLine CL 1000 Bioreactor (Integra). Briefly, 25 × 10^6^ cells were suspended in 15 ml serum-free Hybridoma-SFM medium (Gibco) supplemented with hybridoma mix (2,800 mg/l of d-glucose, 2,300 mg/l of peptone, 2 mM l-glutamine, 1% penicillin/streptomycin, 1% non-essential amino acids, 0.00017% 2-mercaptoethanol) and cultured in the cell compartment separated from the nutrient supply compartment by a semipermeable membrane. The nutrient supply medium was identical to the medium used for the cell compartment. Supernatant containing W6/32 antibody was harvested from the cell compartment following a 7-day culture period. HB-65 and OKT4 hybridomas were cultured in RPMI 1640 medium containing 10% FBS and Iscove’s Modified Dulbecco Medium containing 20% FBS, respectively. FBS concentration was gradually reduced to <5% during the culture period to minimize bovine serum albumin content in antibody preparations.

### HLA Typing and Sequencing

Total genomic DNA was extracted from 5 × 10^6^ cells using the QIAamp DNA Mini Kit (QIAGEN) according to the manufacturer’s instructions. Four-digit typing of HLA class I/II alleles expressed by cell lines was routinely performed at the WIMM HLA typing and Sequencing facility using Sanger sequencing on a 48 capillary ABI-3730 DNA analyzer. To confirm the presence of intact HLA-C*01:02 in CD4.221 DNA, full length HLA-C sequencing was performed to 8-digits resolution using a recently developed in-house mono-allelic sequence-based technique (manuscript in preparation). Briefly, a pair of gene-specific primers was used to amplify the whole HLA-C locus (4.1 kbp), then the product was enzymatically cleaned using ExoSap-IT (Thermo Fisher Scientific). The products were then sequenced in both direction using the Sanger sequencing technique as above. The sequence traces were assembled and analyzed using CLC Main Workbench v7.8.1 (QIAGEN).

### HIV-1 IIIB Propagation

The lab-adapted X4-tropic clade B strain HIV-1 IIIB was obtained from the NIBSC CFAR, then prepared by propagation in C8166 cells as previously described (25). Briefly, C8166 cells were infected with HIV-1 IIIB in a minimal volume of R10 medium for 90 min at 37°C, then resuspended at 3.75 × 10^5^ cells/ml in R10. At day 5 postinfection, virus-containing supernatant was harvested then concentrated by underlaying a 5 ml sucrose cushion (20% sucrose, 0.2 mM EDTA in PBS) then spinning at 70,000 × *g* for 2 h at 4°C as described (29). The virus-containing pellet was resuspended in R10 medium, aliquoted and frozen at −80°C.

### *In Vitro* HIV-1 IIIB Infection and Intracellular p24 Staining

C8166, CD4.221, and T2 cells were infected with HIV-1 IIIB in a low volume of R10 medium at a MOI of 0.1 for 90 min at 37°C. Cells were then resuspended in R10 at a concentration of 3.75 × 10^5^ cells/ml (20 ml total) in upright T175 flasks. To each flask, 20 ml R10 was added on day 1 then on day 2, cells were split 1:2, and 20 ml fresh R10 was added to each flask. At day 3, the percentage of cells productively infected with HIV-1 IIIB was determined by intracellular p24 staining as previously described (30). Briefly, 10^6^ cells were washed in 5 ml PBS, then stained with Live/Dead Aqua fixable dye (Life Technologies). Cells were washed then fixed in 4% paraformaldehyde containing 20 µg/ml lysolecithin for 2 min at room temperature, then permeabilized in cold 50% methanol/PBS for 15 min. Cells were incubated in 0.1% NP-40/PBS for 5 min at 4°C, then stained with FITC-conjugated anti-HIV-1 Gag p24 (clone KC57; Beckman-Coulter) for 15 min at room temperature and finally washed once in 5 ml PBS. Staining data were acquired on a CyAn ADP Analyzer with Summit Software and analysis was performed on FlowJo software version 8.8.6 (FlowJo LLC). HIV-1 IIIB-infected CD4.221, T2, and C8166 cell cultures routinely contained >50% live p24^+^ cells on the day of harvest and lysis.

### Flow Cytometry

To determine HLA-DR expression, cell lines and PBMCs were washed then stained with anti-HLA-DR-PerCP (BD Biosciences, clone L243) for 15 min at room temperature and then washed and fixed in 4% PFA. Staining data were acquired and analyzed as described above.

### Antibody Purification and Resin Preparation

To purify antibodies, supernatant from hybridomas was harvested and cleared at 2,500 × *g* for 25 min at 4°C, then filtered through a 0.2 µm SteriCup Filter (Millipore) and adjusted to pH 8.0 with 1 M Tris. Antibody was captured by addition of 2–3 ml protein A resin (PAS) (Expedeon) suspended in PBS for 30 min at room temperature on an orbital shaker at 100 rpm. Beads were collected by gravity flow through a chromatography column and then washed with 20–30 ml PBS. Captured antibody was eluted by addition of 5 ml 100 mM glycine pH 3.0 to the beads and then 1 ml fractions were collected into tubes containing 50 µl 1 M Tris pH 9.5. Buffer was exchanged for PBS by concentration through a 5 kDa molecular weight cutoff centrifugal filter. Antibody purity was routinely checked by observing the presence of light and heavy chains *via* SDS-PAGE. W6/32 and OKT4 antibody specificity was routinely checked by staining cells known to express, or not express, HLA class I and/or CD4.

Antibody cross-linking was performed as previously described (31). Briefly, 1 mg purified antibody per 0.5 ml PAS was incubated at 4°C for 1 h. Beads were collected by gravity flow through a chromatography column, washed with 15 ml borate buffer (0.05 M boric acid, 0.05 M KCl, 4 mM NaOH), and equilibrated with 15 ml 0.2 M triethanolamine pH 8.2. Antibody was then cross-linked to beads with 15 ml 40 mM dimethyl pimelimidate in 0.2 M triethanolamine pH 8.3 for 1 h at room temperature. The cross-linking reaction was ended by addition of 15 ml ice-cold 0.2 M Tris pH 8.0. Unlinked antibody was removed by flowing through 15 ml citrate buffer pH 3.0 followed by 2–3 washes with 15 ml PBS to neutralize pH.

### HLA class I Immunoprecipitation (IP)

Peptide–HLA class I complex purification was performed as previously described (31). Briefly, approximately 2–3 × 10^8^ uninfected or HIV-1 IIIB-infected cells were harvested for each experiment. Cells were washed once in PBS then pelleted and 1 ml IGEPAL buffer [0.5% IGEPAL 630, 50 mM Tris pH8.0, 150 mM NaCl and 1 tablet cOmplete Protease Inhibitor Cocktail EDTA-free (Roche) per 10 ml buffer] was added per 0.5–1 × 10^8^ cells, and cells were lysed by mixing for 45 min at 4°C. Lysates were cleared by sequential centrifugation steps at 2,000 × *g* for 10 min, then 20,000 × *g* for 15 min at 4°C. Peptide–HLA class I complexes (or CD4 for OKT4 IPs) were captured by incubation of lysate with 1–2 mg antibody coupled to PAS overnight at 4°C on a rotator in a final volume of 15–25 ml. The lysate was flowed through a pre-washed chromatography column, then the beads were consecutively washed with 15 ml wash buffer 1 (0.005% IGEPAL, 50 mM Tris pH 8.0, 150 mM NaCl, 5 mM EDTA), 15 ml wash buffer 2 (50 mM Tris pH 8.0, 150 mM NaCl), 15 ml wash buffer 3 (50 mM Tris pH 8.0, 450 mM NaCl), and 15 ml wash buffer 4 (50 mM Tris pH 8.0). Peptide–HLA complexes were eluted by addition of 3–5 ml 10% acetic acid and collected in 1 ml fractions, and then dried in a vacuum drier.

### Reversed-Phase High Performance Liquid Chromatography (RP-HPLC) Peptide Purification

Reversed-phase high performance liquid chromatography was performed as previously described (31). Briefly, immunoaffinity column-eluted peptide–HLA complexes were resuspended by vortexing and sonication in 120 µl 0.1% trifluoroacetic acid (TFA)/1% acetonitrile and then injected onto a 4.6 mm × 50 mm ProSwift RP-1S column (ThermoFisher Scientific) and eluted using a 500 µl/min flow rate over 10 min from 3 to 30% buffer B (0.1% TFA in acetonitrile) using an Ultimate 3000 HPLC system (ThermoFisher Scientific). Detection was performed using a variable wavelength detector at 280 nm. Odd and even fractions of 500 µl up to 12 min that did not contain β2-microglobulin were combined and dried.

### Enzymatic Protein Digestion for Proteomic Analysis

CD4.221 cells were lysed in IGEPAL lysis buffer, then 10 µg lysate was digested with trypsin. Briefly, cysteine residues were alkylated with 20 mM iodoacetamide for 30 min, then reduced again in 50 mM DTT for 30 min at room temperature. Samples were diluted with 800 µl Milli-Q water, then digested with 200 ng sequencing-grade porcine trypsin (Promega) for 16 h at 37°C. Digested peptides were purified on C18 Sep-Pak Light cartridges and eluted in 65% acetonitrile/0.1% TFA, then dried. Peptides were resuspended in 0.1% TFA/1% acetonitrile for LC-MS/MS analysis.

### LC-Tandem Mass Spectrometry (LC-MS/MS)

#### IP Samples

Purified peptides were resuspended in 20 µl 0.1% TFA/1% acetonitrile then 8 µl were injected onto an Ultimate 3000 HPLC system coupled online to a Fusion Lumos mass spectrometer (ThermoFisher Scientific). Peptides were separated with a 75 µm × 50 cm PepMap RSLC C18 EasySpray column using a linear gradient from 3 to 25% buffer B in buffer A (0.1% FA in water) at a flow rate of 250 nl/min for 60 min. Peptides were introduced to the Fusion Lumos using an EasySpray source. Precursors were selected in top-speed mode within a 2 s cycle time (accumulation time of 120 ms) and an isolation width of 1.2 amu for fragmentation. Higher-energy collisional dissociation (HCD) with a collision energy setting of 28 was performed on the peptides with a charge state of 2–4, while a higher collision energy of 32 was applied to singly charged precursor ions that were selected with lower priority. MS resolution was set at 120,000 and MS^2^ resolution was set at 30,000. All fragmented precursor ions were actively excluded from repeated selection for 30 s.

#### Tryptic Digestion Sample

Tryptic peptides were separated on an Ultimate 3000 HPLC system supplemented with a 75 µm × 50 cm PepMap RSLC C18 EasySpray column using a linear gradient from 2 to 35% buffer B at a flow rate of 250 nl/min for 180 min. Peptides were introduced to the Q-Exactive-HF using an EasySpray source. HCD with a collision energy setting of 28 was performed on the top 15 most abundant precursor ions per MS full scan (injection time of 41 ms) using an isolation width of 1.0 amu. Full MS resolution was set at 120,000 and MS^2^ resolution was set at 15,000. Only peptides with a charge state of 2–5 were isolated and fragmented. All fragmented precursor ions were actively excluded from repeated selection for 81 s.

### Mass Spectrometry Data Analysis

Data was imported into PEAKS 8 software (Bioinformatic Solutions) as .raw files. A database (20,243 entries) containing all annotated human Swiss-Prot entries (current at 10/08/2017) including translations of all six reading frames of the sequenced HIV-1 IIIB genome (GenBank KJ925006) (25) was used for interpretation of MS/MS spectra. Precursor and fragment error tolerances were set at 5 ppm and 0.03 Da, respectively. A false discovery rate (FDR) of 5% (unless otherwise indicated) was defined using parallel decoy database searches. No fixed or variable modifications were set. Peptide spectrum matches were exported as .csv files and peptides <7 amino acids in length were excluded from all analyses. Where two possible peptides differing only by a single leucine to isoleucine residue were identified, both matches were retained in the dataset. Putative HLA class I or II restriction and binding affinity of identified peptides was predicted using the NetMHCcons, NetMHC4.0, or NetMHCII online algorithms (found at http://www.cbs.dtu.dk/services/) (15). Sequence logos of identified peptides were produced using the default settings for Shannon type logos in Seq2logo2.0^1^ (32). Area-proportional Venn diagrams were created using the online BioVenn tool^2^ (33). Gibbs Clustering analysis was performed with the online GibbsCluster2.0 server^3^ using the default settings for MHC class I ligands with 1–5 clusters (34). For comparison to mass spectrometry-acquired data, known peptide ligands for HLA class I alleles were extracted from the Immune Epitope Database (IEDB).^4^ The Los Alamos National Laboratory HIV database QuickAlign Tool^5^ was used to map the location of HIV-1-derived peptides in the HIV-1 genome. Graphs were created using GraphPad Prism 7 software (GraphPad Software Inc.).

## Results

### HLA IP Allows Reproducible Identification of HLA Class I-Bound Peptides

**Figure 1:**
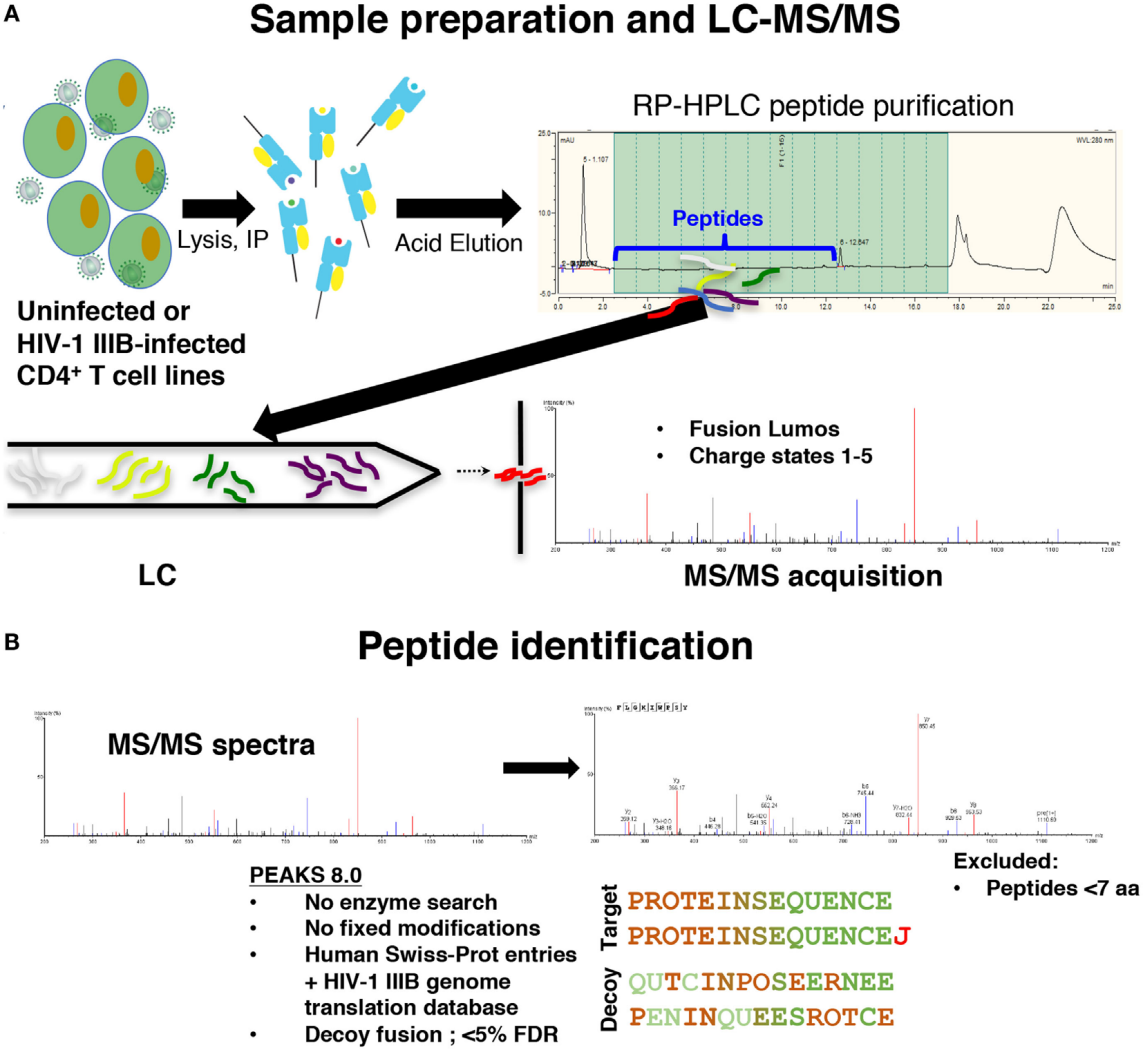
Immunopeptidomics workflow. **(A)** C8166, T2, or CD4.221 cell lines were mock-infected or infected with human immunodeficiency virus type 1 (HIV-1) IIIB, then cell lysates were incubated with the pan human leukocyte antigen class I-specific antibody W6/32, CD4-specific antibody OKT4, or the influenza A NP-specific antibody HB-65 for immunoprecipitation. Peptides were purified by reversed-phase high performance liquid chromatography (RP-HPLC), then LC-MS/MS data were acquired on a Fusion Lumos instrument. **(B)** Peptide identifications were made in PEAKS 8.0 software using a 5% false discovery rate cutoff defined using parallel decoy database searches. Peptides with a length of less than seven amino acids were excluded from all analyses.

To distinguish novel viral peptides presented on HLA class I from potential co-purified contaminants, a careful analysis of the reproducibility and specificity of our immunopeptidomics workflow was required. We immunoprecipitated peptide–HLA class I complexes from cell lysates as previously described (31) (Figure 1). To characterize the reproducibility of the HLA class I IP and LC-MS/MS methods, HLA class I peptides were isolated from uninfected C8166 cells (a CD4^+^ cell line) or C8166 cells infected with the lab-adapted X4-tropic HIV-1 strain IIIB. Replicate peptide samples prepared on separate days (biological replicates) were sequenced by LC-MS/MS. Additionally, data for one HIV-1-infected C8166 peptide sample were acquired in duplicate to address the magnitude of instrumental variability (technical replicates).

**Figure 2:**
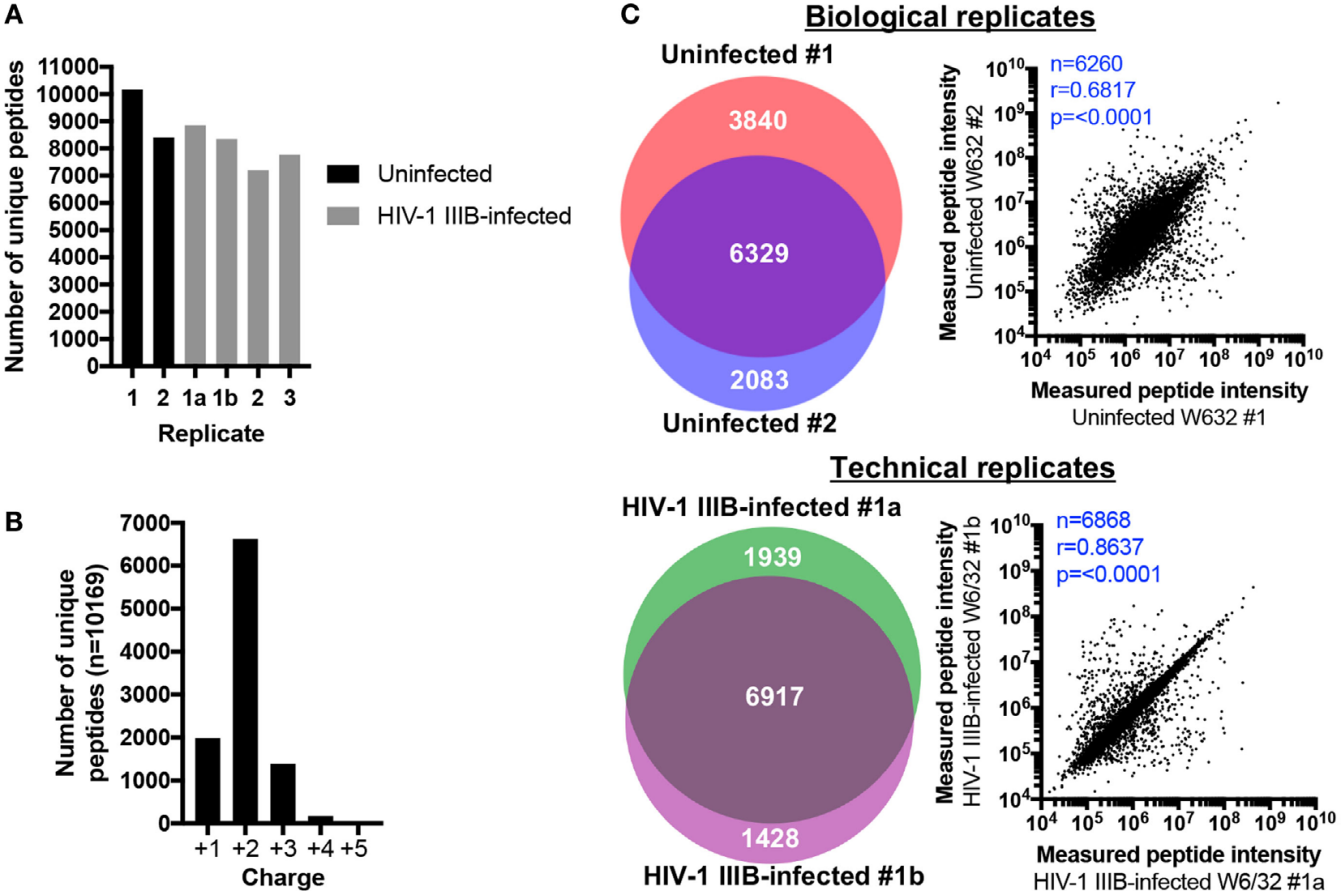
Human leukocyte antigen (HLA) immunoprecipitation allows reproducible identification of HLA class I-bound peptides. HLA class I complexes were immunoprecipitated from either uninfected or human immunodeficiency virus type 1 (HIV-1) IIIB-infected C8166 cells (HLA type: A*01:01, B*08:01, B*44:02, C*05:01, C*07:01) using the W6/32 antibody and then sequenced by LC-MS/MS. **(A)** Summary of the number of unique peptides identified in each biological or technical replicate for uninfected (black bars) or HIV-1 IIIB-infected (gray bars) cells. Samples labeled 1, 2, or 3 are biological replicates and samples labeled 1a and 1b are technical replicates. **(B)** Charge state of identified peptides from uninfected replicate #1. **(C)** *Left*: area-proportional Venn diagrams displaying the numerical overlap in peptide identifications between biological or technical replicates. *Right*: the measured area under the curve (intensity) for peptides shared between biological and technical replicates is shown. Where intensity values were missing for one or both replicates (uninfected biological replicates *n* = 69, HIV-1 IIIB-infected replicates *n* = 49), these peptides were excluded from the analysis. Rho values and *p* values for the Spearman’s rank correlation are shown.

Between 8412-10169 and 7202-8856 peptides were identified in uninfected and HIV-1-infected samples, respectively (Figure 2A). For each sample, about two thirds of peptides were doubly charged and approximately 20% of peptides were singly charged (data for uninfected replicate #1 is shown in Figure 2B as an example). 6,917 peptides were identified in both technical replicates, representing 67.26% of all unique peptides identified across both samples (or 78.11 and 82.89% of the technical replicates 1a and 1b, respectively) (Figure 2C). Regarding biological replicates, 6,329 peptides (51.66% of unique peptides across both replicates, or 62.24% and 75.24% of biological replicates 1 and 2, respectively) were identified in both uninfected replicate #1 and #2 (Figure 2C). Thus, one can reasonably expect that up to 80% of identified peptides will be re-identified in a second LC-MS/MS analysis of the same sample, while up to 70% of peptides will be shared between biological replicates. These data suggest that the HLA IP and LC-MS/MS method is qualitatively reproducible. To determine whether similar quantities of peptides were also measured in replicate samples, the measured intensities of peptides identified in replicate C8166 samples were plotted against each other to determine whether the amount of peptide detected in one replicate predicted the quantity of the same peptide in a repeat measurement. As expected, the measured peptide intensity in one sample was highly predictive of its quantity in a technical replicate (*r* = 0.8637, *p* < 0.0001; Figure 2C). Similarly, the quantity of peptides measured in one biological replicate positively correlated with the peptide intensity in a sample prepared on a different day (*r* = 0.6817, *p* < 0.0001; Figure 2C).

**Figure 3:**
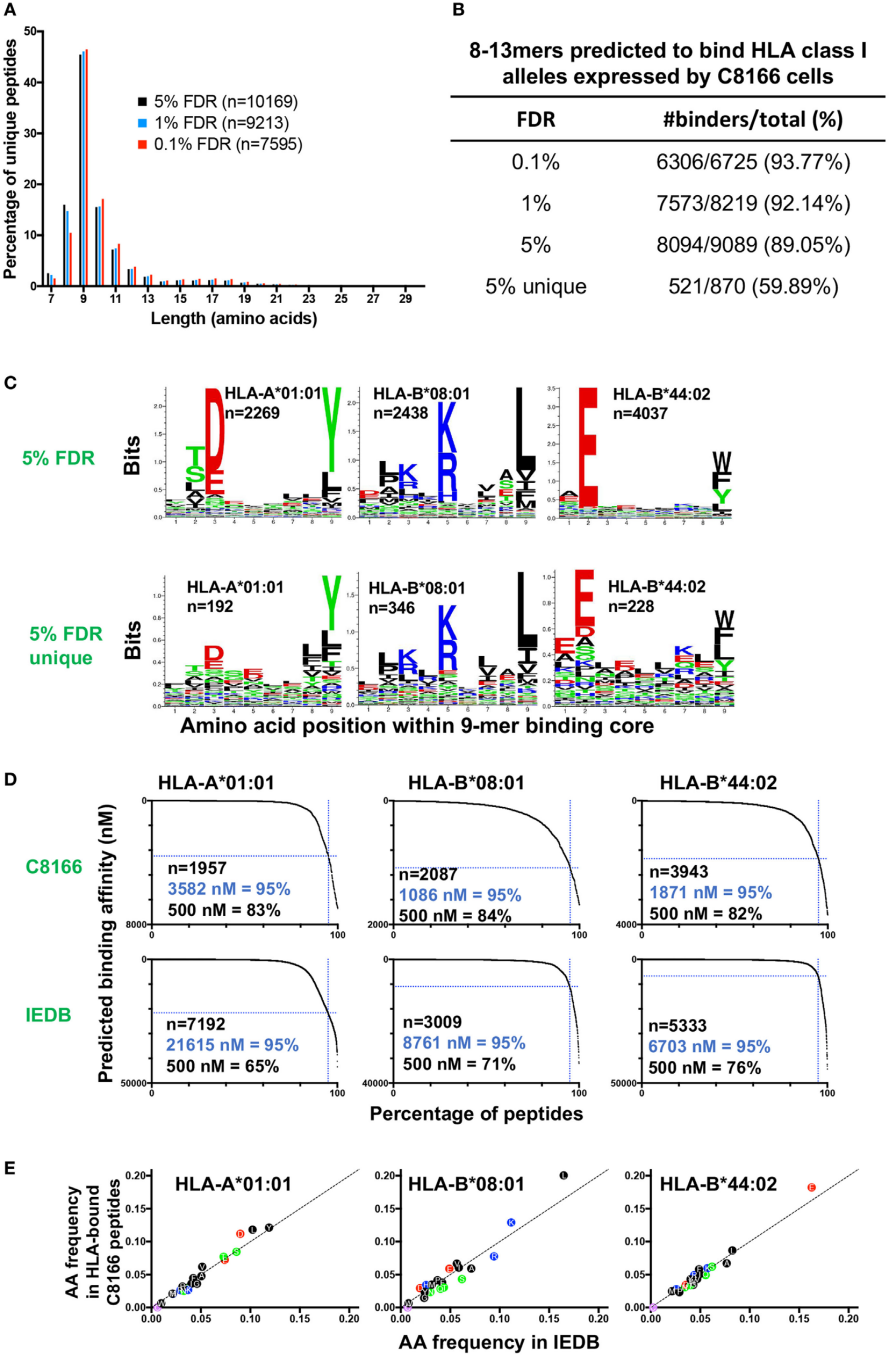
Characteristics of human leukocyte antigen (HLA) class I-bound peptides identified by LC-MS/MS. HLA class I complexes were immunoprecipitated from uninfected C8166 cells (HLA type: A*01:01, B*08:01, B*44:02, C*05:01, C*07:01) using the W6/32 antibody, then peptides were sequenced by LC-MS/MS. **(A)** Length distribution of peptides identified in the uninfected C8166 replicate #1 at false discovery rates (FDRs) of 0.1, 1, and 5%. **(B)** All 8–13-mer peptides identified at the indicated FDRs were tested *in silico* for binding to the HLA class I alleles expressed by C8166 cells using NetMHC4.0. Peptides were classified as “binders” when they were predicted to bind with a higher affinity than 98% of 400,000 randomly generated peptides (i.e., 2% rank or lower). “5% unique” indicates those peptides identified at 5% FDR but not at 1% FDR. **(C)** All 8–13-mers identified at 5% FDR or those identified only at 5% FDR (not at 1% FDR) in the uninfected replicate #1 sample were clustered using the online GibbsCluster algorithm. Each cluster identified by GibbsCluster is represented by a sequence logo, which corresponds to the indicated HLA class I allele expressed by C8166 cells. The number (*n*) of peptides in each cluster is also shown. In the sequence logo, amino acids are represented by their single letter code. The more frequently an amino acid occurs at a position within peptides, the larger the letter is displayed. **(D)** Binding affinity to HLA-A*01:01, HLA-B*08:01, or HLA-B*44:02 was predicted for all 8–13-mers identified at 5% FDR in the uninfected replicate #1 sample (top row) or for all 8–13-mers listed as restricted by each allele in Immune Epitope Database (IEDB) (bottom row). The number of peptides (*n*) is indicated along with the affinity threshold at which 95% of peptides bound or the proportion of peptides, which bound with higher affinity than 500 nM (a limit set in previous iterations of the NetMHC algorithm). **(E)** All 8–13-mer peptides identified at the indicated FDR were tested *in silico* for binding to the HLA class I alleles expressed by C8166 cells using NetMHC4.0. The amino acid frequency for peptides predicted to bind to one of HLA-A*01:01, HLA-B*08:01, or HLA-B*44:02 in the uninfected C8166 replicate #1 was plotted against the amino acid frequency in peptides assigned to each allele in IEDB.

Immunopeptidomic studies have typically utilized parallel target-decoy database approaches to estimate a peptide FDR of between 1 and 5%. In order to determine a FDR suitable for optimizing false positive and negative identification in our immunopeptidomics epitope discovery approach, we compared the peptides identified in the uninfected C8166 cells replicate #1 sample at 0.1, 1, or 5% FDRs. As expected, the most common peptide length was 9 amino acids while relatively few peptides had a length greater than 13 amino acids, a distribution which is typical for HLA class I-bound peptides (Figure 3A). At a highly stringent 0.1% FDR longer peptides (10–13-mers) were favored, whereas a greater proportion of identified peptides were 8-mers at the relaxed 5% FDR (Figure 3A).

Human leukocyte antigen class I alleles bind peptides containing sequence motifs favorable for stabilization of the HLA complex. The extent to which the method specifically sampled HLA class I-bound peptides was estimated by binding affinity prediction using NetMHC. Because HLA class I binding affinity predictions for peptides longer than 13-mers are trained on relatively small datasets and are therefore less reliable than for the more common 9-mers, we limited our analysis to 8–13-mers. At 0.1, 1, and 5% FDRs, 93.77, 92.14, and 89.05% of identified 8–13-mers were predicted to bind the HLA class I alleles expressed by C8166 cells (Figure 3B). When considering peptides only found at the 5% FDR (above the score threshold for 1% FDR), 59.89% of peptides were predicted to bind to HLA class I. Given that a high proportion of peptides identified by the immunopeptidomics approach were predicted to bind to HLA class I, one would expect that amino acids which favor stabilization of the HLA complex would be present at anchor positions within the peptide. We used GibbsCluster to align and cluster all the 8–13-mers identified at 5% FDR or those only identified at 5% FDR into groups with shared sequence motifs, then visualized amino acid enrichment at particular positions using Seq2Logo. Based on the greatest information content (Kullbach Leibler distance) for each number of clusters made, three clusters were found to be optimal for the eluted C8166 8–13-mers identified (Figure 3C). Upon manual inspection and comparison to known binding motifs, these clusters were found to represent ligands for the HLA class I alleles HLA-A*01:01 (homozygous), HLA-B*08:01, and HLA-B*44:02 expressed by C8166 cells (see Table 1 for full HLA type of cell lines used in this study). No distinct HLA-C*05:01 and HLA-C*07:01 (expressed by C8166 cells) clusters could be identified through this method. Examination of peptides identified only at the 5% FDR (not at 1% FDR) revealed similar amino acid enrichment at anchor positions despite lower peptide numbers, indicating that a large number of false negatives occur at 1% FDR as suggested by another study (35). On the basis of these results, we implemented a 5% FDR cutoff for data presented in this study.

**Table 1:** Human leukocyte antigen (HLA) class I and II alleles present in cell lines used in this study.

Cell line	HLA-A	HLA-B	HLA-C	HLA-DRB1	HLA-DRB345	HLA-DQB1
C8166	01:01	08:01	05:01	0301	DR3	02
	01:01	44:02	07:01	0401	DR4	0301
CD4.221	NF	NF	01:02	0101	NF	05
	NF	NF	NF	NF	NF	NF
T2	02:01	51:01	01:02	NF	NF	NF
	NF	NF	NF	NF	NF	NF

*NF, none found*.

A recent study suggested that peptides cataloged in the IEDB include a higher proportion of peptides that display lower-binding affinities to their assigned alleles than peptides identified by LC-MS/MS following HLA IP (36). We used NetMHC to predict binding affinities to HLA-A*01:01, HLA-B*08:01, and HLA-B*44:02 for peptides identified in the uninfected C8166 HLA IP or peptides assigned to each allele in IEDB. For all alleles, peptides identified by LC-MS/MS were predicted to bind with higher affinity than peptides cataloged in IEDB (Figure 3D). These differences were not a result of gross alterations in amino acid frequencies within peptides identified by LC-MS/MS, as frequencies were similar to those reported in IEDB (Figure 3E). Altogether, these data suggest that our immunopeptidomics workflow identifies peptides with higher binding affinity than those identified through alternative methods, which are reported in IEDB.

### CD4.221 Cells Express and Present Peptides on HLA-C*01:02

**Figure 4:**
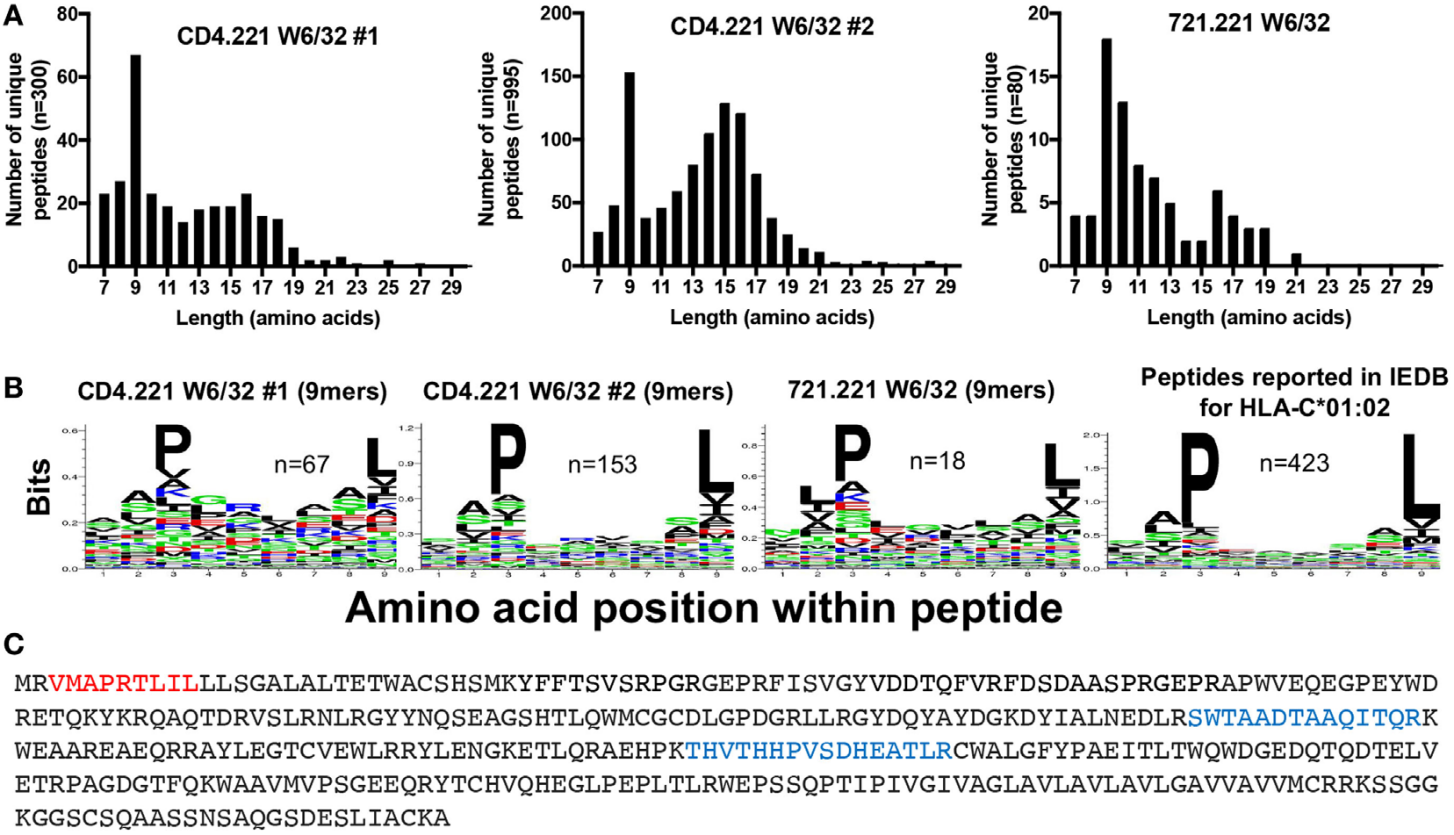
CD4.221 cells express and present peptides on human leukocyte antigen (HLA)-C*01:02. CD4.221 cells were infected with human immunodeficiency virus type 1 IIIB, then lysed and W6/32-conjugated resin was used for immunoprecipitation (IP). Peptides identified were compared to those eluted from W6/32 IPs of 721.221 cells reported by Abelin et al. (36) and peptides reported as HLA-C*01:02-restricted in Immune Epitope Database (IEDB). **(A)** Length distributions of peptides eluted from CD4.221 cells or 721.221 cells. **(B)** Sequence logos of 9-mer peptides eluted from CD4.221 cells, 721.221 cells, or 9-mers reported as C*01:02-restricted in IEDB. **(C)** The protein sequence of the HLA-C*01:02 gene amplified and sequenced from genomic CD4.221 DNA is shown. Highlighted in red is the leader sequence identified in the W6/32 IP from CD4.221 lysates. Highlighted in blue are peptides which were identified in a proteomic analysis of CD4.221 cell lysate and which do not correspond to sequences from HLA-E or HLA-F sequence (which are also expressed by CD4.221 cells).

To gain further insight into the specificity of our immunopeptidomics workflow, we performed HLA class I IPs from the CD4.221 cell line. The 721.221 B lymphoblastoid cell line (parental line to CD4.221 cells) is widely regarded to be HLA class Ia-deficient (only expressing low levels of the non-classical class I molecules HLA-E and HLA-F). It would thus be expected that if the immunopeptidomics workflow solely identifies HLA class I-bound peptides, very few peptides would be detected in W6/32 IPs of CD4.221 cells. However, although the number of unique peptides identified was substantially lower than that identified from C8166 cells, we nonetheless observed a striking enrichment of 9-mers (replicate #1, *n* = 67; replicate #2, *n* = 153) after HLA class I IP from CD4.221 cells (Figure 4A). Analysis of the length distribution of peptides identified in a recent study which performed HLA class I IP from the 721.221 cell line demonstrated a similar enrichment of 9-mers (36). Given that 721.221 cells are known to express HLA-E, we first predicted whether the 9-mers eluted from CD4.221 cells would potentially bind to HLA-E using NetMHC4.0. Indeed, over one-third of the eluted 9-mers were predicted to bind to HLA-E (data not shown). However, on closer examination of the sequence motif of eluted 9-mers, a preference for proline at position 3 and leucine at position 9 was observed in the CD4.221 and 721.221 datasets (Figure 4B). As shown in Figure 4B, these amino acid preferences are reported in IEDB for HLA-C*01:02 ligands.

The parental B cell line 721 was originally determined to express HLA-A*01:01, A*02:01, B*08:01, B*51:01, C*07:01, and C*01:02 (37). These alleles were presumed to be lost or not expressed in the 721.221 cell line as a consequence of γ-ray-induced mutation in the HLA gene region, despite the detection of full length HLA-C*01:02 by Southern blotting (38). We noted that the HLA class I signal sequence peptide “VMAPRTLIL” was present in both the CD4.221 and 721.221 HLA class I IPs (Figure 4C). This signal sequence, which is presented by HLA-E under normal physiological conditions (39), is encoded by the HLA-A*01:01 or HLA-C*01:02 genes. To confirm the presence of the HLA-C*01:02 gene in the CD4.221 cell line, we used specific PCR primers to amplify the HLA class Ia genes from DNA, then performed full length gene sequencing. No sequence amplification was observed when using HLA-A or HLA-B-specific primers. However, HLA-C-specific primers recovered a sequence identical to that of the known HLA-C*01:02:01 allele (the amino acid sequence is shown in Figure 4C), with the exception of an intronic G to T mutation at nucleotide 1004. HLA-C*01:02 (but not HLA-A or HLA-B) was also amplified from the parental cell line 721.221 (data not shown). To provide further evidence for HLA-C*01:02 expression at the protein level, we examined the CD4.221 proteome following tryptic digest of CD4.221 lysate. In addition to peptides unique to the non-classical molecules HLA-E and HLA-F, two peptides unique to HLA-B/HLA-C alleles were identified which could therefore be assigned to the sole classical HLA gene present, HLA-C*01:02 (Figure 4C). Thus, in contrast to the widely held belief that .221 cells are HLA class Ia negative, HLA-C*01:02 is expressed (albeit at low levels) and binds a limited number of peptides in 721.221/CD4.221 cells.

### Some Non-Specific Pull-Down of HLA Molecules Occurs in Membrane Protein IPs

**Figure 5:**
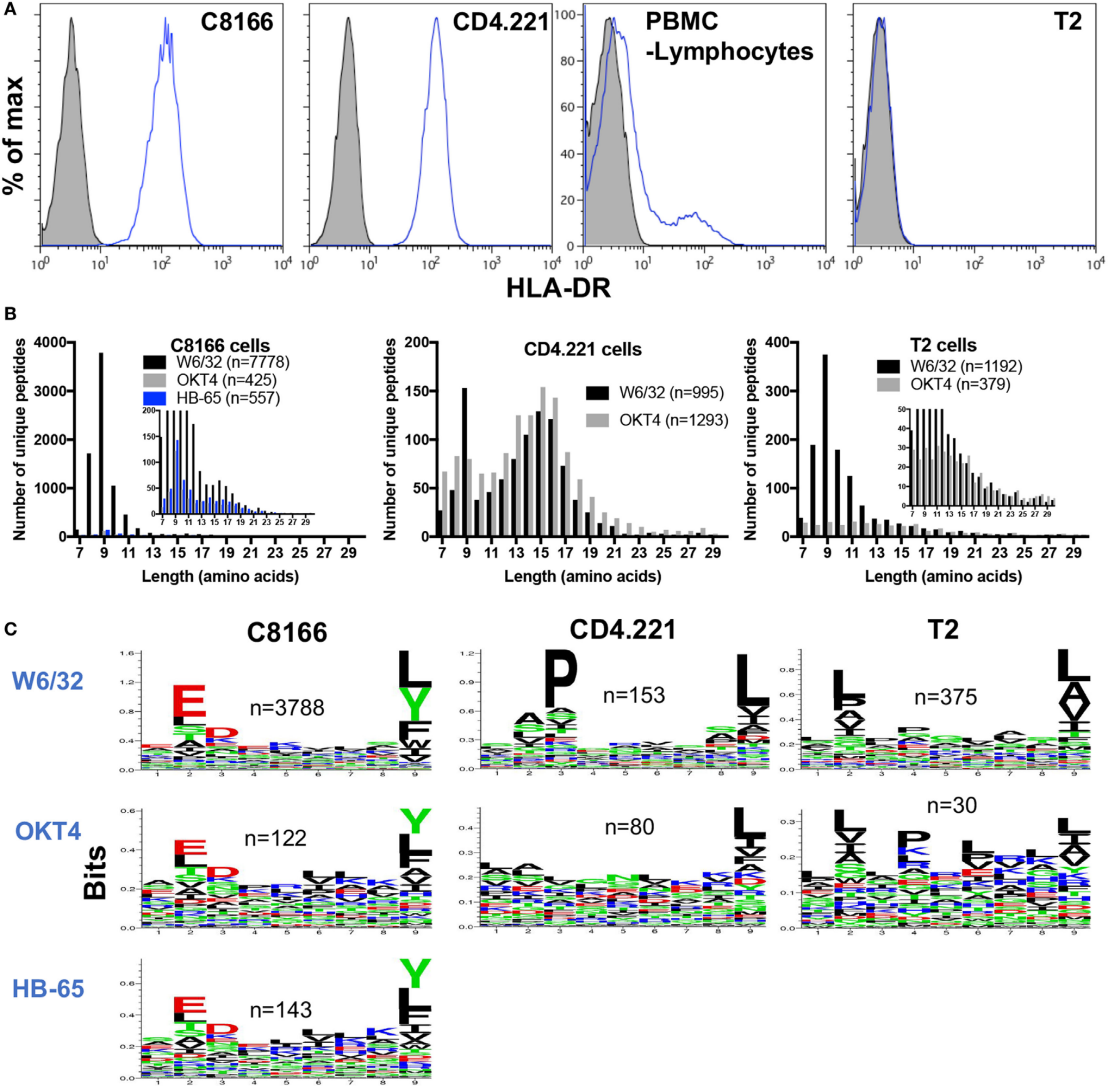
Some non-specific pull-down of human leukocyte antigen (HLA) molecules occurs in membrane protein IPs. **(A)** Flow cytometry plots showing HLA-DR expression was measured by cell surface staining with L243 (blue lines) of C8166, CD4.221, peripheral blood mononuclear cells, or T2 cells. Doublets and dead cells were removed from analyses. Unstained controls are indicated by the gray filled histograms. Plots are gated on live single cells. **(B)** CD4.221, T2, or C8166 cells were infected with human immunodeficiency virus type 1 IIIB for 3 days, then cells were lysed and IP was performed with W6/32-, HB-65- (C8166 only), or OKT4-conjugated resin. Purified peptides were sequenced by LC-MS/MS. The length distributions of peptides eluted from each cell lysate with the indicated antibody are shown. *Inset*: same data scaled on a reduced *y*-axis. Data shown are representative of two separate experiments. **(C)** Sequence logos of 9-mers identified from the same samples as in **(B)**.

Although inspection of peptides eluted from HLA class I IPs of C8166 or CD4.221 cells revealed specific enrichment for 8–13-mers containing anchor residues favorable for HLA class I stabilization, a sizeable proportion of identified peptides were longer than 13 amino acids in length (819 peptides for C8166 uninfected replicate #1 and 544 peptides for CD4.221 replicate #2) (Figures 3A and 4A). The majority (68% for C8166, 85% for CD4.221) of these extended peptides were 14–18 amino acids in length. Furthermore, 559 of the 819 (68%) peptides with a length greater than 13 amino acids in the C8166 sample formed “nested sets” with overlapping sequences extended at either N- or C-terminus. These nested sets were comprised of between 2 and 44 peptides from 110 different proteins. This length distribution in addition to the prevalence of nested sets are features which are reminiscent of HLA-DR-restricted peptides reported in previous immunopeptidomic studies (40–42). As shown by cell surface staining with a HLA-DR-specific antibody, C8166 cells, CD4.221 cells, and a proportion of the peripheral blood lymphocytes from a healthy donor (activated T cells are known to express HLA-DR) expressed surface HLA-DR (Figure 5A). T2 cells [in which the HLA class II gene region is deleted (43)] were used as a negative control. Therefore, we hypothesized that many of the extended peptides eluted from C8166 or CD4.221 cells resulted from co-purification of HLA class II complexes in HLA class I IPs.

Given that some HLA class II molecules seemed to be non-specifically pulled down in HLA class I IPs (despite a lack of cross-reactivity with HLA-DR of the W6/32 antibody used for class I IP), we predicted that a low level of HLA class I and II complexes would also be co-isolated following IPs of other membrane-associated proteins. We used the CD4-specific antibody OKT4 in parallel with W6/32 to ascertain the level of HLA class II complex co-purification in membrane protein IPs. We also performed IPs with an antibody directed against a protein not expressed in C8166 cells (the influenza A nucleoprotein-specific antibody HB-65) as a control to determine the level of non-specific binding of C8166 lysate components to antibody-linked-resin. Furthermore, to examine the extent of HLA class II complex co-purification, peptides were eluted from membrane IPs (W6/32 or OKT4-conjugated resin) of HLA class II-deficient T2 cells. In parallel W6/32 and OKT4 IPs from CD4.221 lysate, 995 and 1,293 unique peptides were identified, respectively (Figure 5B). The HLA class II size distribution centered around 15-mers was present in both W6/32 and OKT4 IPs. However, 9-mers were not enriched in the OKT4 IP to the same extent as in the W6/32 IP. In contrast, although considerably lower numbers of peptides were identified relative to the W6/32 IP (*n* = 7,778), the OKT4 (*n* = 425), and HB-65 (*n* = 557) IPs from the C8166 lysate showed an enrichment for 9-mers in addition to longer nested sets, indicating non-specific pull-down of both HLA class I and II complexes by membrane protein IP. Peptides eluted from the T2 W6/32 IP (*n* = 1,192) were enriched in 8–13-mers, but not for 14–16-mers as observed for HLA class II-expressing CD4.221 and C8166 cells. Of the 184 peptides of at least 14 amino acids in length in the T2 cell sample, 139 peptides originated from 20 proteins, many of which are reported to be abundant contaminants non-specifically associating with resin (44) (such as keratin, actin, ribosomal proteins; data not shown).

If HLA class I complexes were co-purified during OKT4/HB-65 IPs, one would also expect to observe enrichment of amino acids favorable for HLA class I stabilization at anchor positions within the peptide. We observed such an enrichment for anchor residues within 9-mers identified in both the OKT4 and HB-65 IPs from C8166 cells (Figure 5C). Furthermore, enrichment for anchor residues was also seen in OKT4 IPs from T2 cells or, to a lesser extent, CD4.221 cells, despite the considerably lower numbers of 9-mers detected. Altogether, these data indicate that HLA class I and/or II peptides can be non-specifically co-isolated when membrane protein IPs are performed.

### Some HIV-1-Derived Peptides Are Non-Specifically Co-Purified During Membrane Protein IPs From HIV-1-Infected Cells

**Figure 6:**
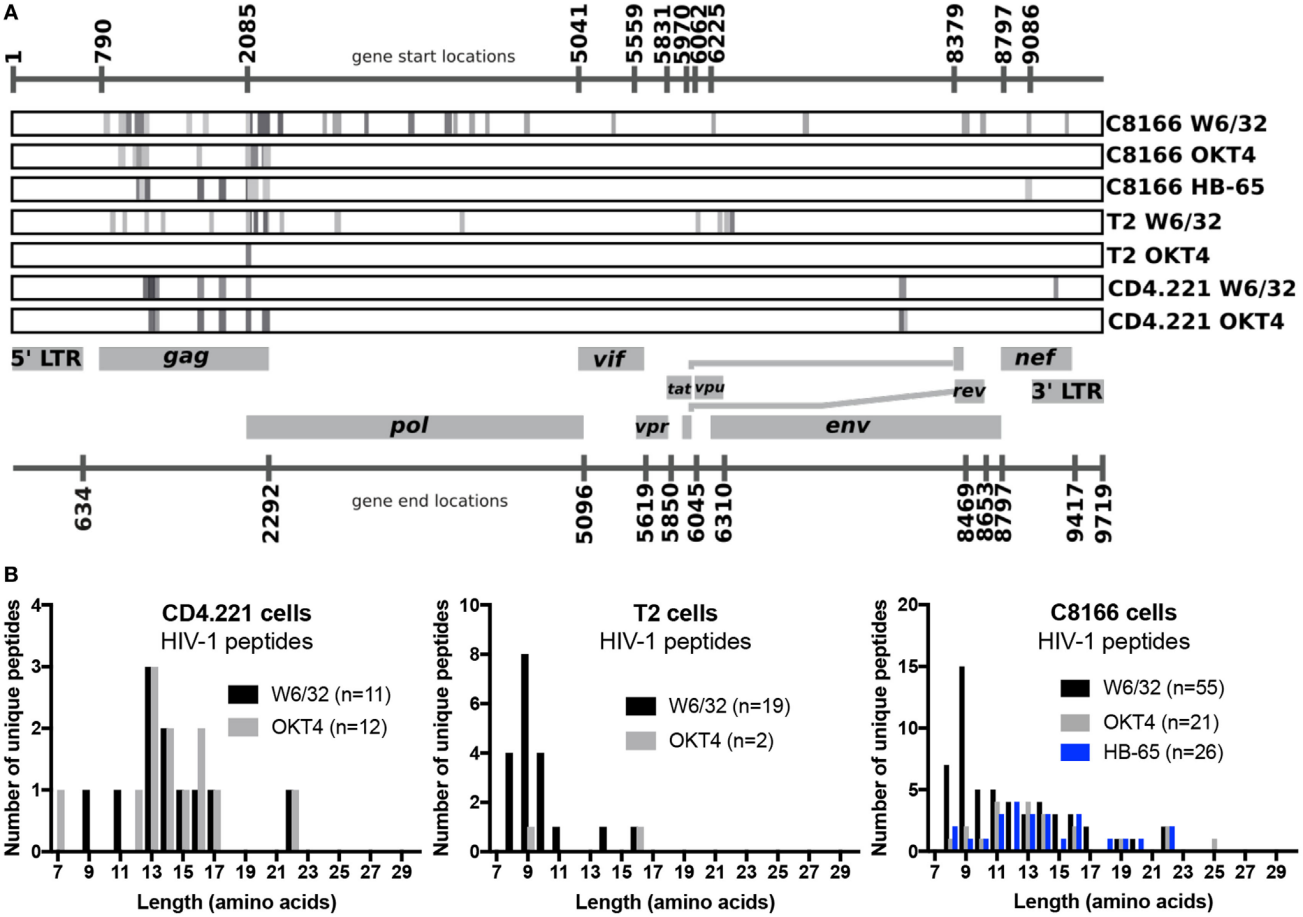
Human immunodeficiency virus type 1 (HIV-1) peptides are non-specifically co-purified from HIV-1-infected cells by human leukocyte antigen class I immunoprecipitation (IP). C8166, CD4.221, and T2 cells were infected with HIV-1 IIIB, then lysed and W6/32-, HB-65- (C8166 only), or OKT4-conjugated resin was used for IP. Purified peptides were sequenced by LC-MS/MS. **(A)** Schematic summary of HIV-1 peptides identified in each sample tested in this study. Numbers above and below the schematic indicate the start and end nucleotide locations of the nine conventional genes encoded by HIV-1 reference strain HXB2. Peptides (or nested sets of peptides) identified in each sample (indicated on the right of each rectangular box) are illustrated as a gray stripe. Diagram adapted from the Los Alamos National Laboratory HIV-1 QuickAlign tool. **(B)** Length distributions of HIV-1 peptides identified in each sample.

Since we showed that HLA class II complexes could be co-purified with HLA class I IPs, we examined the possibility that some of the HIV-1-derived HLA class I ligands identified in previous immunopeptidomics studies or those identified in this study were non-specifically co-isolated. When HIV-1 IIIB-infected CD4.221 cell lysate was immunoprecipitated with W6/32 and OKT4, 11 and 12 HIV-1-derived peptides were identified, respectively (Figure 6A; Table 2). These peptides predominantly originated from the abundantly expressed HIV-1 structural protein Gag. Strikingly, the majority of these peptides were 13–17-mers (Figure 6B; Table 2). Furthermore, they included a nested set (four overlapping peptides) encompassed by the VDRFYKTLRAEQASQEV species that has been previously described as a dominant CD4^+^ T cell epitope restricted by multiple HLA-DR alleles including HLA-DR1 (45). In contrast, the 19 HIV-1-derived peptides identified in the W6/32 IP of HIV-1 IIIB-infected T2 cell (HLA class II-deficient) lysate were primarily 8–11-mers from Gag, Pol, Env, and Vpu (Figure 6B; Table 3). Similarly, 39 of 55 HIV-1 peptides identified in the W6/32 IP from HIV-1-infected C8166 lysate were 8–13-mers while many of the remaining extended peptides contained the canonical C-terminal anchor tyrosine (Y) residue for HLA-A*01:01 (Figure 6B; Table 4). However, the Gag-derived peptides FLGKIWPSYKGRPGNF (which corresponds to the entire Gag p1 sequence) and PIVQNIQGQMVHQAISPRTLNA were identified in both CD4.221 and C8166 membrane IPs (Table 2), indicating non-specific co-isolation of HLA class II-restricted or membrane-associated peptides. These peptides were also observed in the parallel OKT4 and HB-65 IPs from C8166 lysate, where approximately half of the identified peptides were greater than 14 amino acids in length. Therefore, these data show that HLA class II-restricted or membrane-associated non-HLA-bound HIV-1 peptides are non-specifically co-purified with HLA class I IP preparations.

**Table 2:** List of human immunodeficiency virus type 1 (HIV-1) peptides identified in CD4.221 cell lysate membrane protein IPs.

			CD4.221 cells		T2 cells		C8166 cells		
Peptide	Length	HXB2 location^a^	W6/32	OKT4	W6/32	OKT4	HB-65 OKT4^c^	W6/32	Human leukocyte antigen (HLA) restriction^d^
**Detected in CD4.221, T2, and C8166 samples**									

FLGKIWPSYKGRPGNF	16	Gag_433–448_	**Y**^b^	**Y**	**Y**	**Y**	**Y**	**Y**	–

**Detected in CD4.221 and/or C8166 samples only**									

PIVQNIQGQMVHQAISPRTLNA	22	Gag_133–154_	**Y**	**Y**			**Y**	**Y**	^e^
AEAMSQVTNSATIM	14	Gag_364–377_	**Y**	**Y**			**Y**		DR1
PLTSLRSLFGNDPSSQ	16	Gag_485–500_		**Y**			**Y**	**Y**	–
TGHSSQVSQNY	11	Gag_122–132_	**Y**				**Y**		–
VVEEKAFSPEVIP	13	Gag_158–170_	**Y**	**Y**					**DQ5**
VVEEKAFSPEVI	12	Gag_158–169_		**Y**					**DQ5**
VDRFYKTLRAEQASQEV	17	Gag_297–313_	**Y**	**Y**					**DR1**
RFYKTLRAEQASQEV	15	Gag_299–313_	**Y**	**Y**					**DR1**
RFYKTLRAEQASQE	14	Gag_299–312_	**Y**	**Y**					**DR1**
RFYKTLRAEQASQ	13	Gag_299–311_	**Y**	**Y**					**DR1**
GIKQLQARILAVE	13	Env_572–584_	**Y**	**Y**					^e^
ERYLKDQ	7	Env_584–590_		**Y**					–
SLVRPDLSL	9	Cryptic	**Y**						–

*^a^Amino acid location within HIV-1 protein aligned to reference strain HXB2*.

*^b^“Y” indicates the peptide was detected in the sample noted at the top of the column*.

*^c^Peptides were found in at least one of HB-65 and/or OKT4 IPs of C8166 cell lysates*.

*^d^Previously described HLA restrictions detailed in the Los Alamos National Laboratory HIV Immunology Database are indicated in bold font. HLA restrictions predicted by NetMHCII for the HLA class II alleles expressed by the CD4.221 cells are in normal typeface. Hyphens (–) indicate indeterminate HLA restriction*.

*^e^CD4^+^ T cell responses have been observed to these peptides, but the restricting HLA allele was not mapped*.

**Table 3:** List of human immunodeficiency virus type 1 (HIV-1) peptides identified in T2 cell lysate membrane protein IPs.

			T2 cells		C8166 cells		
Peptide	Length	HXB2 location^a^	W6/32	OKT4	HB-65 OKT4^c^	W6/32	Human leukocyte antigen (HLA) restriction^d^
**Peptides identified in T2 and C8166 samples**							

FLGKIWPSY	9	Gag_433–440_	**Y**^b^	**Y**	**Y**	**Y**	A*01:01
FLGKIWPSYK	10	Gag_433–441_	**Y**			**Y**	A*02:01

**Peptides identified in T2 samples only**							

RFAVNPGL	8	Gag_43–50_	**Y**				–
SLYNTVATL	9	Gag_77–85_	**Y**				A*02:01
AISPRTLNA	9	Gag_146–154_	**Y**				E*01:01
MLKETINEEA	10	Gag_200–209_	**Y**				–
ALGPAATL	8	Gag_336–343_	**Y**				–
FLGKIWPS	8	Gag_433–439_	**Y**				A*02:01
TAPPEESFRSG	11	Gag_456–466_	**Y**				–
YPLTSLRSL	9	Gag_484–492_	**Y**				–
SLFGNDPSSQ	10	Gag_491–500_	**Y**				–
GGIGGFIKV	9	Pol_104–112_	**Y**				–
FSVPLDEDFRKYTA	14	Pol_271–284_	**Y**				–
IVTDSQYAL	9	Pol_650–658_	**Y**				–
QPIQIAIV	8	Vpu_2–10_	**Y**				–
HAPWDVDDL	9	Vpu_74–82_	**Y**				–
VWKEATTTL	9	Env_44–52_	**Y**				–
KAYDTEVHNV	10	Env_59–68_	**Y**				–

*^a^Amino acid location within HIV-1 protein aligned to reference strain HXB2*.

*^b^“Y” indicates the peptide was detected in the sample noted at the top of the column*.

*^c^Peptides were found in at least one of HB-65 and/or OKT4 IPs of C8166 cell lysates*.

*^d^Previously described HLA restrictions detailed in the Los Alamos National Laboratory HIV Immunology Database are indicated in bold font. HLA restrictions predicted by NetMHC or NetMHCcons for the HLA class I alleles expressed by the T2 (or C8166 in the case of FLGKIWPSY) cells are in normal typeface. Hyphens (–) indicate indeterminate HLA restriction*.

**Table 4:** List of human immunodeficiency virus type 1 (HIV-1) peptides identified in C8166 cell lysate membrane protein IPs.

Peptide	Length	HXB2 location^a^	HB-65 OKT4^b^	W6/32	Human leukocyte antigen (HLA) restriction^d^
GQLQPSLQTGSEERRSLYNTVATLY	25	Gag_62–86_	**Y**^c^		A*01:01, B*44:02, DR4, DRB3
GSEERRSLYNTVATLY	16	Gag_71–86_		**Y**	A*01:01, B*44:02, DR4
QTGSEERRSLY	11	Gag_69–79_		**Y**	A*01:01
GSEERRSLY	9	Gag_71–86_		**Y**	A*01:01, C*07:01
SKKKAQQAAADTGHSSQVSQNY	22	Gag_111–132_	**Y**	**Y**	A*01:01, C*05:01, DQ3
KKAQQAAADTGHSSQVSQNY	20	Gag_113–132_	**Y**	**Y**	A*01:01, C*05:01, DQ3
KAQQAAADTGHSSQVSQNY	19	Gag_114–132_	**Y**	**Y**	A*01:01, C*05:01, DQ3
AQQAAADTGHSSQVSQNY	18	Gag_115–132_	**Y**		A*01:01, C*05:01, DQ3
AAADTGHSSQVSQNY	15	Gag_118–132_	**Y**	**Y**	A*01:01, C*05:01, DQ3
AADTGHSSQVSQNY	14	Gag_119–132_	**Y**	**Y**	A*01:01, C*05:01, DQ3
ADTGHSSQVSQNY	13	Gag_120–132_	**Y**	**Y**	A*01:01, DQ3
DTGHSSQVSQNY	12	Gag_121–132_	**Y**	**Y**	A*01:01, DQ3
HSSQVSQNY	9	Gag_124–132_		**Y**	A*01:01
AEQASQEVKNW	11	Gag_306–316_	**Y**	**Y**	B*44:02
GKIWPSYKGRPGNF	14	Gag_435–448_	**Y**		–
KIWPSYKGRPGNF	13	Gag_436–448_	**Y**		–
WPSYKGRPGNF	11	Gag_438–448_	**Y**		–
PSYKGRPGNF	10	Gag_439–448_	**Y**	**Y**	–
SYKGRPGNF	9	Gag_440–448_	**Y**	**Y**	–
YKGRPGNF	8	Gag_441–448_	**Y**		–
LQSRPEPTAPPFLQ	14	Gag_449–462_	**Y**		–
SRPEPTAPPFLQ	12	Gag_451–462_	**Y**		–
SRPEPTAPPEESFRSG	16	Gag_451–466_	**Y**		–
PLTSLRSLFGND	12	Gag_485–496_	**Y**		–
PLTSLRSLFGNDP	13	Gag_485–497_	**Y**	**Y**	DR4
TSLRSLFGNDPSSQ	14	Gag_487–500_		**Y**	DR4
LRSLFGNDPSSQ	12	Gag_489–500_	**Y**		DR4
RSLFGNDPSSQ	11	Gag_490–500_	**Y**	**Y**	DR4
WQRPLVTIKIGGQ	13	Pol_62–74_	**Y**		DRB4
WQRPLVTIKIGGQL	14	Pol_62–75_	**Y**		DRB4
FLKEKGGL	8	Nef_90–97_	**Y**	**Y**	B*08:01
KAAVDLSHFLKEK	13	Nef_82–94_	**Y**		–
RLRPGGKKKYKL	12	Gag_20–31_		**Y**	B*08:01
RPGGKKKYKLK	11	Gag_22–32_		**Y**	–
EIKDTKEAL	9	Gag_93–101_		**Y**	B*08:01
EIYKRWII	8	Gag_260–267_		**Y**	B*08:01
ETTTPPQKQEPIDKELY	17	Gag_468–484_		**Y**	A*01:01
TTPPQKQEPIDKELY	15	Gag_470–484_		**Y**	B*44:02
EEMSLPGRW	9	Pol_90–98_		**Y**	–
LVDFRELNK	9	Pol_229–237_		**Y**	–
KSVTVLDVGDAY	12	Pol_259–270_		**Y**	–
VTVLDVGDAY	10	Pol_261–270_		**Y**	A*01:01
VLDVGDAY	8	Pol_263–270_		**Y**	A*01:01
FSVPLDEDFRKY	12	Pol_271–282_		**Y**	A*01:01, DR3, DQ2
PLDEDFRKY	9	Pol_274–282_		**Y**	A*01:01
EELRQHLLRW	10	Pol_358–367_		**Y**	B*44:02
IAEIQKQGQGQWTY	14	Pol_481–494_		**Y**	B*44:02, DRB4
AEIQKQGQGQWTY	13	Pol_482–494_		**Y**	B*44:02
AEIQKQGQGQW	11	Pol_482–492_		**Y**	B*44:02
YVDGAANRETKLGKAGY	17	Pol_596–612_		**Y**	A*01:01, DQ3
GAANRETKLGKAGY	14	Pol_599–612_		**Y**	–
LTNTTNQKTELQAIY	15	Pol_624–638_		**Y**	A*01:01
NQKTELQAIY	10	Pol_629–638_		**Y**	–
SESELVNQI	9	Pol_668–676_		**Y**	B*44:02
SAGIRKIL	8	Pol_708–715_		**Y**	B*08:01
HTDNGSNF	8	Pol_829–836_		**Y**	A*01:01, C*05:01
LADQLIHLY	9	Vif_102–110_		**Y**	A*01:01, C*05:01
LADQLIHL	8	Vif_102–109_		**Y**	C*05:01
ISERILSTY	9	Rev_55–63_		**Y**	A*01:01
SAEPVPLQL	9	Rev_67–75_		**Y**	C*05:01
RVKEKYQHL	9	Env_2–10_		**Y**	B*08:01, C*07:01
FTDNAKTII	9	Env_277–285_		**Y**	A*01:01, C*05:01, C*07:01
ELKNSAVSL	9	Env_806–814_		**Y**	B*08:01
RLAFHHVA	8	Nef_188–195_		**Y**	–

*^a^Amino acid location within HIV-1 protein aligned to reference strain HXB2*.

*^b^Peptides were found in at least one of HB-65 and/or OKT4 IPs of C8166 cell lysates*.

*^c^“Y” indicates the peptide was detected in the sample noted at the top of the column*.

*^d^Previously described HLA restrictions detailed in the Los Alamos National Laboratory HIV Immunology Database are indicated in bold font. HLA restrictions predicted by NetMHC, NetMHCcons or NetMHCII for the HLA class I and II alleles expressed by the C8166 cells are in normal typeface. Hyphens (–) indicate indeterminate HLA restriction*.

## Discussion

Due to significant advances in the sensitivity of state-of-the-art instrumentation, mass spectrometry-based immunopeptidomic approaches hold promise to elucidate the nature of the complex repertoire of peptides presented to the immune system by diverse cell types. In recent years, immunopeptidomic methodologies have been employed to discover, among others, novel vaccine candidates for infectious diseases as well as cancer neoantigens (16, 46). In-depth analysis of immunopeptidomic data has also revealed the unexpected findings that peptides derived from out-of-frame translation and “non-coding” genomic areas (47) or from proteasome-catalyzed peptide splicing may constitute a large portion of the immunopeptidome (48). Given their increasing application in immunological studies, definition of the reproducibility and specificity of immunopeptidomic workflows is important for the discovery and validation of *bona fide* epitope targets for vaccines or immunotherapies.

Here, we assessed the reproducibility of our immunopeptidomics workflow using biological and technical replicates of peptide samples eluted from C8166 cells. The observed 50 and 70% overlap in peptide identifications between biological and technical replicates is comparable to results reported in a prior paper despite the use of additional peptide length restrictions, binding affinity cutoffs, and score thresholds in that study (49). The reproducibility between replicates could be further improved (at the cost of false negatives) by using a more stringent 1% FDR rather than the 5% FDR used here. A comparison of FDR cutoffs showed that more 8-mers were identified at 5% FDR, whereas longer peptides (≥10 amino acids) were favored at more stringent FDR cutoffs. Notably, HLA-B*08:01 (expressed by C8166 cells) is restricted in length preference, with octamers commonly identified (50). Therefore, stringent FDR cutoffs may underestimate the frequency of peptides restricted by alleles such as HLA-B*08:01. In line with this, peptides identified at the 5% FDR cutoff only (not at FDR of 1%) predominantly conformed to the HLA-B*08:01 binding motif following Gibbs clustering (Figure 3C).

Analysis of eluted ligands also revealed that the peptides identified here tended to have a higher predicted affinity for HLA class I than peptides listed in IEDB, suggesting that IP-based immunopeptidomics is biased toward detection of higher affinity ligands. Prior estimates suggest that the HLA IP method recovers a small proportion of the total HLA class I complexes (28). Hence, we speculate that HLA complexes loaded with lower affinity ligands are relatively less stable and are lost during the IP procedure.

We found that the immortalized B cell line CD4.221 (and hence the parental 721.221 cell line) presents peptides on endogenously expressed HLA-C*01:02. 721.221 cells were presumed to be HLA class Ia-deficient following their original description (38), as a relatively low level of staining with the pan HLA class I-specific antibody W6/32 was attributed to expression of the non-classical molecule HLA-E. The underlying reason for the level of HLA-C*01:02 expression in the 721.221 cell line being so low is not clear, although influences from microRNA regulation, structural properties of the binding cleft and intrinsic complex stability/instability may be involved, as suggested to explain the variation observed in the expression of other HLA-C alleles (51–53). Many of the peptides putatively restricted by HLA-C*01:02 eluted from CD4.221 cells in this study were also predicted to bind to HLA-E by NetMHC. Experimental evidence for an overlap in peptide binding specificities is limited; however, the vaccinia virus epitope D8L was previously reported to bind with almost identical affinities to both HLA-C*01:02 and HLA-E (54). Given that the pan class I-specific W6/32 and HLA-C-reactive DT9 antibodies both recognize HLA-E, careful interpretation is warranted when 721.221 cells are peptide-pulsed and these antibodies are used as a readout. HLA-C*01:02 expression in 721.221 cells may also have implications for NK cell assays using 721.221 cells as targets (peptide-pulsed or otherwise) while investigating particular killer immunoglobulin receptor–HLA interactions. Finally, immunopeptidomic studies of 721.221 HLA class I transfectants should subtract “background” peptides from untransfected cells to avoid corruption of resulting binding motifs with HLA-C*01:02-bound peptides. This may have particular relevance for alleles such as the non-classical HLA-G, which binds a limited repertoire of peptides and displays a similar binding motif to that of HLA-C*01:02 (55).

By performing IPs from CD4.221, T2, and C8166 cells with a CD4-specific antibody, we showed that both HLA class I and/or II molecules were non-specifically pulled down in membrane protein IPs. A similar phenomenon was noted in a recent study eluting peptides from 721.221 cells transfected with membrane-bound HLA-C*06:02, where a large proportion of peptides greater than 14 amino acids in length without canonical anchor residues were detected (56). When the authors expressed truncated soluble HLA-C*06:02, co-purification of extended peptide species was reduced, although not completely ablated. Accordingly, the level of specificity of peptides detected may vary depending on the individual immunopeptidomics protocol. Another recent study of >130,000 peptides derived from cell lines and tumor tissue found that 3.4% of the total peptides were identified in both HLA class I and II IPs (57). Although the authors speculate that these peptides (which conform to a mixture of HLA class I and II length distributions) may arise from the cross-presentation pathway, it is possible that some HLA class II complexes were co-purified with HLA class I IPs and *vice versa* in this setting. It is clear that HLA class II complexes were a dominant source contaminating our HLA class I IPs; however, it is possible that non-classical HLA class I molecules are also co-isolated. Recently, HLA-F was shown to accommodate binding of particularly long peptides through an open-ended groove as a result of a R62W substitution in the HLA-F heavy chain (58). Peptides eluted from soluble HLA-F produced by 293T cells were of variable length, and preferred acidic glutamate (E) and aspartate (D) or basic lysine (K) residues in the C-terminal anchor position. Although enrichment of C-terminal D, E, or K residues was not observed in the current study within long peptides eluted from C8166, T2, or CD4.221 cells, we cannot rule out that some of the extended peptides we detected were bound to HLA-F.

The non-specific elution of HLA class I/II-binding peptides in the “irrelevant antibody” HB-65 IP from C8166 cell lysates was unexpected. The epitope for this influenza NP-specific antibody has, to our knowledge, never been mapped. We noted a low level of C8166 cell surface staining by HB-65 by flow cytometry (data not shown). Therefore, we reason that a low level of HB-65 cross-reactivity with unknown membrane proteins may explain the peptide number and length profile being similar to that observed in the CD4 IP.

Considering all our data together, we speculate that the cell lysis method used here generates very small “membrane fragments,” which contain several host/viral proteins, resulting in the latter being pulled down during the IP step in addition to the molecule of interest. This finding has clear implications for HLA epitope discovery, as (1) peptides eluted from HLA class II molecules could potentially be erroneously assigned as HLA class I-restricted or as novel CD8^+^ T cell vaccine candidates and (2) when purifying particular molecules using allele-specific antibodies, other HLA class I/II allele complexes may contaminate IP preparations. An important goal for the field of immunopeptidomics will be the exploration of a variety of lysis methods, detergent concentrations, or extra sonication steps in order to obtain a purer membrane-associated HLA preparation. Future studies should compare different detergents at varying concentrations, as well as how sonication methods might affect yield of pure HLA complexes from cell lysates. This optimization may have to strike a balance between purity and yield, as high concentrations of detergent could cause HLA class I complex dissociation.

Particular species of unusually long HIV-1 Gag peptides in HLA class I IP preparations have been identified in three separate prior studies that used different methodologies to identify novel CD8^+^ T cell targets in HIV-1-infected cells: HLA IP from whole cells (25), secreted HLA-A*11:01 IP (26) or MAE from the cell surface (27). Many of these peptides cannot be simply untrimmed species bound to endoplasmic reticulum-associated HLA class I molecules, as they were also identified using the acid elution and secreted HLA methodologies. Examples of N- or C-terminally extended sets of HLA class I ligands have previously been reported in the literature. For example, HLA-B*57:01 has been shown to bind nested sets of N-terminally extended HIV-1 peptides with register shifts such that the N-terminus protrudes out of the groove (59). A recent study of HLA-A*02:01 ligands in *Toxoplasma gondii*-infected cells showed that C-terminally extended peptides were eluted (the HLA-A*02:01-binding motif was at the N-terminus), and a crystal structure demonstrated that a peptide extended by one lysine at the C-terminus could displace the Tyr84 residue at the end of the binding groove, thus making the groove “open-ended” (60). Another binding mode of long *T. gondii* peptides to HLA-A*02:01 was shown by Remesh et al. whereby negatively charged amino acids induced a “Lys149 lift” at the end of the binding groove to allow extensions of over eight amino acids on average (61). Although the frequency of these extensions in nature is unclear, a recent preprint article (62) used existing immunopeptidomic datasets to reveal the presence of C-terminal extensions in several HLA class I alleles (for example, up to ~6% of 10mers were predicted to contain C-terminal extensions for the common HLA-A*03:01 allele). However, whether a similar phenomenon can explain the bias toward detection of extended HIV-1 peptides in the aforementioned immunopeptidomic studies is uncertain. Although some long Gag peptides were recognized by HIV-1^+^ patient PBMC in ELISPOT assays in these studies, whether the IFNγ-producing cells were CD4^+^ or CD8^+^ was not explored. Additionally, no peptide fine mapping was performed to determine whether smaller optimal-length peptides within the long sequence could stimulate the same PBMC (many of the long peptides derive from highly immunogenic regions containing 9-mer epitopes restricted to various HLA alleles^6^). Furthermore, Yaciuk et al. used an *in vitro* HLA-A*11:01 peptide binding assay to show that many of the long peptides identified in all three studies (e.g., AM14; AEAMSQVTNPATIM, SF13; SRPEPTAPPEESF; and PQ16; PLASLRSLFGSDPSSQ) showed minimal or no capacity to replace a fluorescent peptide standard on HLA-A*11:01 (26). Given that the HLA IP method is biased toward peptides with high affinity for HLA class I, it is surprising that such long peptides with low affinity for HLA-A*11:01 would also be present in HLA class I peptide samples from multiple donors of diverse HLA types. Many peptides are thought to bind two or more HLA class I molecules (63), but unusually long peptides binding promiscuously across many donor HLA class I types would be unprecedented. Overall, whether these long peptides are really HLA class Ia-bound thus seems questionable.

Our data clearly show that long HIV-1 Gag peptides are non-specifically co-purified with membrane CD4 or HLA class I IPs prepared from lysates of HLA class Ia-low CD4.221 cells. One potential explanation could be that these peptides are HLA-E-restricted, thus explaining their presence in samples of diverse HLA types (as HLA-E is highly conserved across human populations). However, we did not observe HLA-E stabilization by a subset of these HIV-1 peptides (data not shown) and 9-mers are the main peptide length reported to bind HLA-E experimentally (64) or by computational modeling studies (65). Alternatively, the long Gag peptides may be presented by the non-classical HLA-F molecule. However, we did not detect the majority of these extended peptides in membrane protein IPs from T2 cells, which are known to express HLA-F (66). Given that T2 cells are devoid of HLA class II, it is, therefore, likely that these peptides are HLA class II-restricted. In fact, several of the long peptides have previously been reported as CD4^+^ T cell epitopes restricted by HLA-DRB1*01:01 (e.g., VDRFYKTLRAEQASQEV) or HLA-DQB1*05:01 (e.g., VVEEKAFSPEVIP) recognized by HIV-1^+^ patients (45, 67). Other peptides without a known HLA restriction were shown to stimulate CD4^+^ T cells *ex vivo* (68, 69) (PIVQNIQGQMVHQAISPRTLNA and GIKQLQARILAVE), or were predicted to bind to the HLA class II alleles expressed by CD4.221 cells (AEAMSQVTNSATIM).

The Gag peptide FLGKIWPSYKGRPGNF was detected in all samples tested. The presence of this peptide may be explained by the inherent properties of Gag for membrane association and proteolytic processing. By virtue of its co-translational N-terminal myristoylation, Gag associates with membranes where it forms nascent virions incorporating *pol* gene products such as integrase, reverse transcriptase, and viral protease. The maturation process of the virion requires the protease to cleave the precursor Gag (pr55) sequentially into MA, CA, p2, NC, p1, and p6 fragments (70). The p2 (AEAMSQVTNATIM) and p1 (FLGKIWPSYKGRPGNF) fragments generated by this process were among the long HIV-1 Gag peptides identified here and in the three previous immunopeptidomic studies of HIV-1-infected cells (25–27). Thus, it is conceivable that proteolytic products of Gag are associated with membranes, virions, or other compartments within a HIV-1-infected cell and are co-purified in HLA class I IPs. The incorporation of HLA class I (and II) into virions (71) supports this hypothesis, as IP of semi-intact virions or Gag-containing membranous vesicles, then acid elution would liberate Gag fragments from the membrane. Nested sets of these fragments may be a result of differential proteolytic processing or degradation during sample collection. Rucevic et al. demonstrated that the p15 precursor peptides were degraded by cellular cytosolic extracts to form species identical to those observed in HLA class I IPs (27). It is possible that other viral peptides co-purify with HLA class I IP preparations (unusually long Pol peptides were also noted), but this phenomenon may be most noticeable in the case of HIV-1 Gag as it is one of the most abundant proteins produced within infected cells. On this basis, we conclude that some of the long HIV-1 Gag peptides are non-specific contaminants found in HLA class I IP preparations and result from either (1) binding to HLA class II molecules which co-purify with HLA class I immunoprecipitate or (2) membrane-associated proteolytically processed Gag fragments associating with HLA class I IP preparations. This result is of great significance, as the incorporation of these long HIV-1 Gag peptides into candidate vaccines on the basis that they would potentially elicit CD8^+^ T cell responses in all individuals may not enhance the overall immunogenicity of such a vaccine. Thus, on the basis of these data, we believe it is critical that investigators test the “background” of their individual immunopeptidomics protocols and validate candidate epitopes (especially unusually long peptides) before incorporating them into vaccines or immunotherapeutic strategies.

When taking into account peptides eluted non-specifically during the IP step, we report the first dataset of TAP-independent peptides presented by HIV-1-infected cells (Table 3). The majority of these peptides were not previously described and could have utility for HIV-1 vaccine design in CD8^+^ T cell-inducing viral vectors which inhibit TAP (e.g., CMV). Moreover, in addition to novel HIV-1-derived HLA class I-restricted peptides with canonical lengths (8–12-mers), we also observed numerous N-terminally extended sets of long peptides predicted to bind to HLA-A*01:01 expressed by C8166 cells (Table 4). A prior study investigating the repertoire of peptides presented by HLA-A*01:01 revealed a striking tolerance for peptides of up to 18 amino acids in length (72). Whether the unusually long HIV-1-derived potential HLA-A*01:01 peptides identified here represent intermediate untrimmed species present in the endoplasmic reticulum is unknown, as no study to our knowledge has quantified the relative proportion of HLA class I immunoprecipitated from intracellular or surface membrane sources by W6/32 IP. Because C8166 and CD4.221 cells express divergent HLA class II alleles, we cannot exclude the possibility that some of the extended peptides are HLA class II-restricted. Nonetheless, it would be interesting to determine whether such long N-terminally extended sets of HIV-1 peptides stabilize HLA-A*01:01 complexes and elicit distinct T cell responses.

In conclusion, this study has shown that by carefully examining the specificity of the immunopeptidomics methodology, non-specifically co-isolated peptides can be excluded and *bona fide* HLA class Ia-restricted peptides can be identified. Our findings have important implications for the interpretation of results from MS-based immunopeptidomics datasets, and will help to improve the accuracy of identification of pathogen and tumor-derived epitopes of potential use for T cell-inducing vaccine design.

## Author Contributions

TP conducted experiments, analyzed data, and prepared the manuscript. AN prepared W6/32 antibody stocks. AN and NT acquired LC-MS/MS data. AEK prepared OKT4 antibody stocks. L-MY performed amplification and sequencing of the HLA-C gene in CD4.221 DNA. TP, BMK, NT, and PB contributed to study design and data interpretation. All authors were involved in the drafting of the manuscript. The authors declare no conflict of interest.

## Conflict of Interest Statement

The authors declare that the research was conducted in the absence of any commercial or financial relationships that could be construed as a potential conflict of interest.

## Footnotes

^1^http://www.cbs.dtu.dk/biotools/Seq2Logo/index.php (Accessed: August 18, 2017).

^2^http://www.biovenn.nl/ (Accessed: August 16, 2017).

^3^http://www.cbs.dtu.dk/services/GibbsCluster/ (Accessed: August 13, 2017).

^4^http://www.iedb.org/ (Accessed: August 13, 2017).

^5^https://www.hiv.lanl.gov/content/sequence/QUICK_ALIGNv2/QuickAlign.html (Accessed: August 20, 2017).

^6^http://www.hiv.lanl.gov/content/immunology/maps/ctl/Gag.html (Accessed: August 20, 2017).
